# Impacted Chicken Bone in the Laryngopharynx: A Case Report

**DOI:** 10.1155/2011/593504

**Published:** 2011-01-20

**Authors:** Tamer A. Mesallam

**Affiliations:** ^1^Communication and Swallowing Disorders Unit, ENT Department, College of Medicine, King Saud University, Riyadh 11411, Saudi Arabia; ^2^Communication and Swallowing Disorders Unit, Otolaryngology Department, College of Medicine, Minoufiya University, Shebin Al-Koum 32511, Egypt

## Abstract

*Objective*. To describe a rare case of an impacted large foreign body (chicken bone) in the laryngopharynx. *Case Report*. A 28-years-old man presented with pain in the neck of 5 days duration. The patient gave a history of severe choking sensation while eating chicken. Laryngoscopic examination revealed a linear whitish large chicken bone impacted in the left pyriform fossa. The bone was removed under local anesthesia with the guidance of telescopic laryngeal examination. *Conclusion*. This paper describes impaction of a large chicken bone in the hypopharynx in an adult male patient and its removal with guided telescopic laryngeal examination.

## 1. Introduction

Impaction of ingested foreign bodies in the upper aerodigestive tract is a common emergency in the Otolaryngology practice [1–3]. Foreign body impaction commonly occurs in children, mentally challenged, intoxicated adults, and in some manual professions such as electricians, fishermen, and carpenters who hold small items between their teeth while they are working [4]. Presentation of impacted foreign body varies according to its site and size. Usually, patients present with respiratory distress, change in voice quality, or swallowing problems that are preceded by a vigorous or severe attack of choking and coughing [5, 6]. This paper describes impaction of a large chicken bone in the hypopharynx in an adult male patient and its removal with guided telescopic laryngeal examination.

## 2. Case Report

A 28-year-old man presented with severe neck pain mainly on the left side with painful swallowing for 5 days. The patient initially had asked for medical advice at a primary health care facility and was treated with antibiotics and anti-inflammatory drugs, but there was no improvement and the pain was increasing in severity. On detailed history, the patient reported that he had an attack of choking and coughing, while he was eating chicken during dinner one day before the onset of symptoms. 

**Figure 1:**
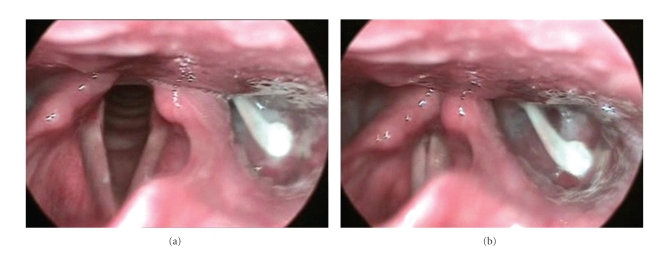
The impacted chicken bone is clearly identified in the left pyriform fossa; (a) laryngeal view during respiration, (b) laryngeal view during phonation.

On examination, there was a low-grade fever and tenderness in the left side of the neck, and the pain was exaggerated by swallowing attempts. A telescopic laryngeal examination was performed. Frothy saliva was detected accumulating in the left pyriform fossa. After giving the patient some few sips of water, the examination was repeated. A white linear chicken bone was seen impacted at the left pyriform fossa with a relatively free broad base pointing anteriorly, and the other tapering end was impacted in the posterior wall of the pyriform fossa (Figure 1). To rule out development of a retropharyngeal abscess, anteroposterior and lateral X-ray views of the neck were performed for the patient. Apart from the chicken bone that was poorly visualized in the lateral X-ray view of the neck, there was no evidence of retropharyngeal abscess formation. 

**Figure 2:**
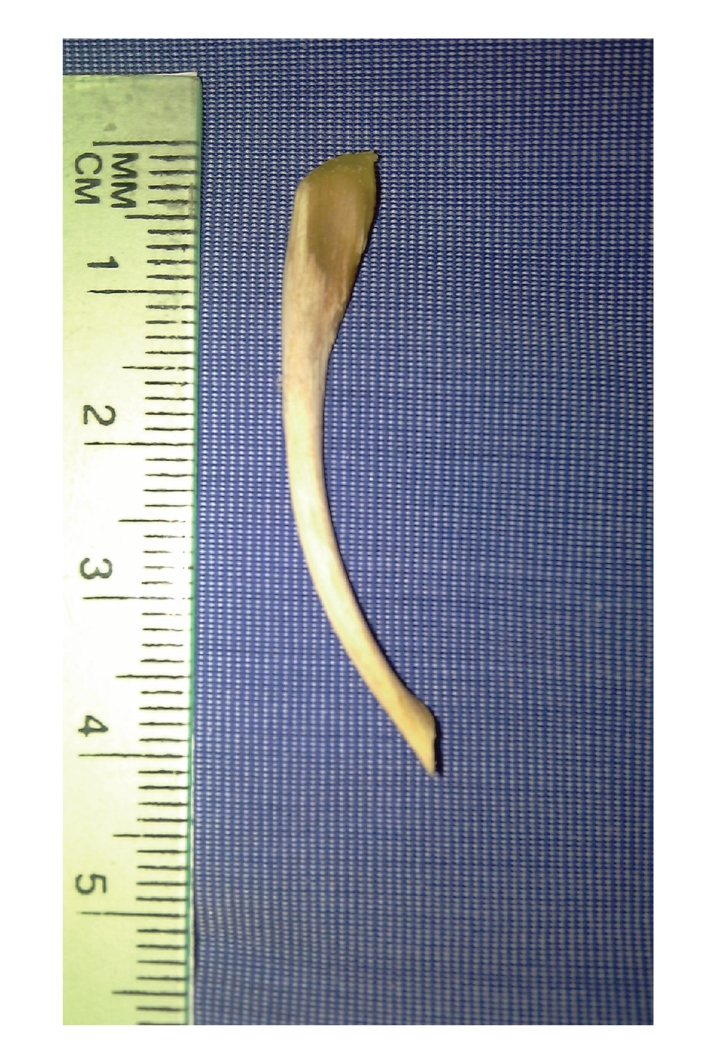
The chicken bone which was almost 4-cm-long after being successfully removed from the hypopharynx.

The bone was removed in an office-based setting under guidance of video laryngeal rigid telescopic examination. In this procedure, a video laryngeal examination was carried out using a 70-degree rigid telescope, 3CCD camera, LCD monitor, and a light source. The patient was seated in the upright position with his head supported from the back. Using a cotton swab soaked with Xylocaine 10% and held by a Magill's forceps, the patient's oro- and hypopharynx were adequately anesthetized. The patient was instructed to protrude his tongue and hold it by his hand with a piece of gauze. The telescope was held by the physician's left hand and introduced carefully into the oropharynx. When the foreign body was identified in the left pyriform fossa and clearly visualized on the monitor, it was carefully removed by a curved laryngeal forceps held by the right hand of the physician (Figure 2). After that, the patient was prescribed oral antibiotics in the form of Amoxycillin-Clavulanic acid (1 gm b.i.d. for 10 days) to control possible infection and an analgesic and anti-inflammatory drug in the form of Diclofenac Sodium (50 mg b.i.d. for 10 days). Then, he was discharged with a scheduled followup appointment after 7 days. On the followup visit, the patient was completely improved with disappearance of the constitutional symptoms, no pain or tenderness on the neck and normal swallowing function.

## 3. Discussion

Foreign body impaction in the larynx is mainly accidental in nature and usually presents as a respiratory emergency that requires urgent intervention to save the patient's life [4]. The clinical presentation may vary according to the site and size of the foreign body. With a large object impacted in the laryngeal vestibule, an acute presentation of severe coughing, choking, hoarseness, and gagging is frequently seen. Occasionally, the patient may not be able to speak, gesticulates wildly, and presents with stridor and cyanosis [7]. However in the current case, the examination revealed the impaction of the chicken bone in the left pyriform fossa. Accordingly, the presenting complaint of the patient was pain in the neck more related to swallowing. Meanwhile, there were no symptoms of respiratory distress or vocal quality impairment. 

Usually, most impacted foreign bodies in the larynx and hypopharynx are removed by direct endoscopy under general anesthesia [8]. Indirect fiberoptic endoscopy is employed in some patients who have a sensitive gag reflex. This is usually performed either through a working channel in the endoscope or with the help of simultaneous transoral forceps. This technique makes some patients uncomfortable, and one must gain experience in operating the flexible nasopharyngoscope and the transoral foreign body forceps simultaneously. In addition, when using a flexible endoscope with a working channel, technical difficulty remains problematic and precise control is hard to achieve because of a poor surgical view and difficulty in manipulating the forceps [3, 9]. 

In this paper, adequate local anesthesia to the oropharynx and the hypopharynx was achieved that made introducing the rigid telescope and the instrument (laryngeal forceps) through the oropharynx less likely to induce a gag reflex. With the advantage of better magnification of the laryngeal and hypopharyngeal view that the rigid laryngeal telescope provides, the bone was clearly identified and demonstrated on the monitor. The bone was carefully removed in an office-based setting using a curved laryngeal forceps. This paper shows that with local anesthesia to the oro- and hypopharynx, a foreign body in the hypolarynx can be comfortably removed in an ambulatory setting under the guidance of video laryngeal telescopic examination.
